# Parkinsonism as a Complication of Bariatric Surgery

**DOI:** 10.3889/oamjms.2015.121

**Published:** 2015-11-24

**Authors:** Walaa A. Kamel, Jasem Y. Al Hashel, Ayman Kilany, Samira Altailji

**Affiliations:** 1 *Neurolgy, Ibn Sina Hospital, Kuwait*; 2 *Beni-suef University, Egypt*; 3 *Kuwait University, Kuwait*; 4 *National Research Centre, Children with Special Needs, Egypt*; 5 *Nuclear Medicine, Ibn Sina Hospital, Kuwait*

**Keywords:** parkinsonism, vitamin deficiency, complication, bariatric surgery, Kuwait

## Abstract

**BACKGROUND:**

The association between Parkinsonism and BS has already been reported in only three patients worldwide.

**CASE SUMMARY:**

We report a 39-years old Kuwaiti female who presented with parkinsonian features and mononeuropathy (carpal tunnel syndrome) 3 years after a vertical sleeve gastrectomy operation.

**CONCLUSION:**

We conclude that with the increasing popularity of bariatric surgery, clinicians will need to recognize and manage neurologic complications that may appear soon after or years to decades later. Thorough evaluation is essential for any patient who has undergone bariatric surgery and develops neurologic symptoms.

## Introduction

Bariatric Surgery (BS) is now considered to be an effective long-term treatment of patients with morbid obesity [1]. Consequently, there has been a significant growth in the number of bariatric operations performed in the past decade. A variety of neurologic complications of bariatric surgery are well documented in the literature [2], many secondary to malabsorption of essential minerals and/or vitamins [3, 4].

In this report we describe one patient who developed parkinsonian manifestations after undergoing bariatric surgery. Our patient was not being followed for development of nutritional deficiencies after surgery.

## Case presentation

A 39-year-old woman underwent a sleeve gastrectomy procedure for morbid obesity, with negative family history of neurological diseases, and her postoperative course was complicated by mild vomiting (antiemetics not given), no postoperative vitamin assay and she lost almost 60 kg in 18 months, she had not attended a nutrition clinic postoperatively and no vitamin supplementation was given. Three years after the operation, she presented with a history of right hand tremor and right UL rigidity and pain. On examination, the patient is fully conscious, normal mood and mentality, resting and postural tremors affected the right hand, cogwheel rigidity and bradykinesia in right upper and lower limbs, decreased arm swing on the right, with normal postural reflexes, normal extra ocular muscles, normal eye movement, normal other general and neurological examinations apart from tenderness in the right elbow joint.

Initial laboratory investigations showed microcytic hypochromic anemia, normal biochemical and electrolytes, negative workup for Wilson disease. Vitamin assay showed low Vitamin D3 (52.6 nmol/l, normal range 75-250), low Vitamin B1 (4.18 ng/ml, normal range (20-100), low Vitamin A (31 mcg/dl, normal range (38-98), low Vitamin B12 (163.7 pg/ml, normal range 180-900), normal copper, normal vitamin B2, normal folate, normal vitamin B6.

**Figure 1:**
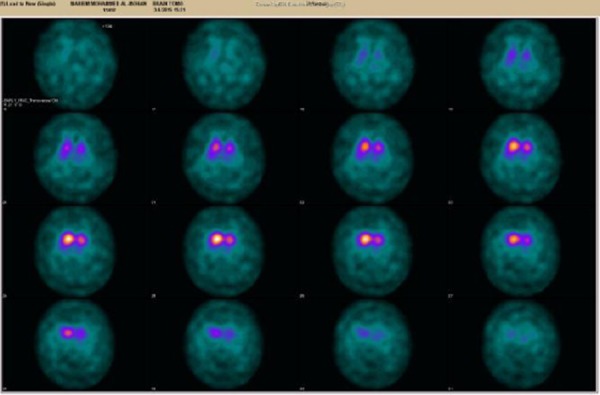
*DAT scan: reduced activity in LT putamen and caudate nuclei*.

Magnetic resonance imagings [MRI] were unremarkable, DAT scan with 123-loflupane (2 years after the onset) showing decreased activity in left putamen, and to lesser extent the left caudate (Figure 1). MRI of RT elbow showed effusion in the elbow joint. Nerve conduction studies (NCS) showed right mild carpal tunnel syndrome (CTS). Normal ultrasound on right median and ulnar nerves (no entrapment). With correction of her vitamin deficiencies and starting her on pramipexole 0.25 mg TDS she starts to improve regarding the tremors and rigidity.

## Discussion

Complications related to the central or peripheral nervous system or both may be seen in approximately 5% to 16% of patients after surgery for peptic ulcer disease or obesity [5]. Neurologic complications following bariatric surgery can involve any level of the neuraxis. Particularly important for functioning of the nervous system are the B-group vitamins (vitamin B12, thiamine, niacin, pyridoxine, folate), vitamin E, copper, and vitamin D. Commonly recognized neurologic complications related to bariatric surgery include encephalopathy, optic neuropathy, myelopathy or myeloneuropathy, radiculoplexus neuropathy, polyneuropathy, and mononeuropathy [6, 7].

Previous report by Fang et al., 2010 [8] described two patients who developed Parkinsonism after undergoing bariatric surgery. Case I: patient who developed encephalopathy and acute Parkinsonism following BS after 6 months. A trial of levodopa led to marked and sustained improvement in the tremor and rigidity. Case II described by Fang et al., 2010 [8]: a patient who presented with course tremors and bradykinesia (6 years after the operation), and administration of a course of levodopa is being considered.

Fabiani et al., 2014 [9] reported a 42-year-old female patient submitted to BS. During the first 2 months the patient presented severe vomiting treated with domperidone. Approximately 4 months later she started with mild rest tremor, bradykinesia and micrograph. The patient was administered levodopa/benserazide (100/25 mg tid) and presented clinical improvement with the treatment. The temporal course of neurologic complications after the procedure is variable and can occur in the immediate perioperative period, short term (within the first year), or in the long-term. It is usually difficult to determine whether a complication is due to deficiency of a single or multiple vitamins. Moreover, micronutrient analysis is neither uniform nor consistent, and these patients have not been characterized clinically. How nutritional deficiency contributes to development of parkinsonism after bariatric surgery is not clear. Recent population studies have cited a possible role of vitamin D insufficiency in the cause of Parkinson disease [10, 11]. Further studies are needed to determine the potential role of vitamin D in pathogenesis of Parkinsonism related to bariatric surgery.

A variety of neurologic complications of bariatric surgery are well documented in the literature, many secondary to malabsorption of essential minerals and/or vitamins [12].

A bariatric procedure essentially reduces the size of the stomach by 90%, leading to early satiety and reduced intake of food. Deficiency in lipid-soluble vitamins after bariatric procedures can occur from a variety of mechanisms. Dumping syndrome, which induces an increase in bowel movements and diarrhea; blind loop syndrome; and functional exocrine insufficiency decrease lipid uptake and result in steatorrhea. In addition, a decrease in gastric acidity and shorter food contact time limit the absorption of nutrients [13]. We also suggest another explanation of developing parkinsonism following BS in our patient is that the patient was already prone to develop parkinsonism but BS may accelerate the condition.

The outcome of neurologic deficits that develop after bariatric surgery is variable and depends on the severity and duration of malnutrition, for post bariatric procedure close laboratory monitoring is recommended [14]. Long term follow-up, dietary counselling and clinical and laboratory evaluations every 6 months are required. In these patients indefinite use of vitamin supplements is recommended [15].

In conclusion, prevention, diagnosis, and treatment of neurologic disorders related to bariatric surgery are necessary elements of lifelong care after bariatric surgery. Preoperative nutritional deficiencies need to be identified and treated. Long-term follow-up with dietary counselling is important and the need for biochemical surveillance of nutritional status after surgery. Routine supplementation of vitamins and minerals may be a cost-effective strategy for preventing neurologic complications in these patients.
